# Systematic Evaluation of Tyrosine Kinase Inhibitors as OATP1B1 Substrates Using a Competitive Counterflow Screen

**DOI:** 10.1158/2767-9764.CRC-24-0332

**Published:** 2024-09-23

**Authors:** Thomas Drabison, Mike Boeckman, Yan Yang, Kevin M. Huang, Peter de Bruijn, Mahesh R. Nepal, Josie A. Silvaroli, Anika T. Chowdhury, Eric D. Eisenmann, Xiaolin Cheng, Navjotsingh Pabla, Ron H.J. Mathijssen, Sharyn D. Baker, Shuiying Hu, Alex Sparreboom, Zahra Talebi

**Affiliations:** 1 Division of Pharmaceutics and Pharmacology, College of Pharmacy, Comprehensive Cancer Center, The Ohio State University, Columbus, Ohio.; 2 Division of Medicinal Chemistry and Pharmacognosy, College of Pharmacy, The Ohio State University, Columbus, Ohio.; 3 Department of Medical Oncology, Erasmus MC Cancer Institute, University Medical Center Rotterdam, Rotterdam, the Netherlands.

## Abstract

Although the primary elimination pathway for most tyrosine kinase inhibitors (TKI) involves CYP3A4-mediated metabolism, the mechanism by which these agents are brought into hepatocytes remains unclear. In this study, we optimized and validated a competitive counterflow (CCF) assay to examine TKIs as substrates of the hepatic uptake transporter OATP1B1. The CCF method was based on the stimulated efflux of radiolabeled estradiol-17β-glucuronide under steady-state conditions in HEK293 cells engineered to overexpress OATP1B1. Of the 62 approved TKIs examined, 13 agents were identified as putative substrates of OATP1B1, and pazopanib was selected as a representative hit for further validation studies. The transport of pazopanib by OATP1B1 was confirmed by decreased activity of its target VEGFR2 in OATP1B1-overexpressing cells, but not cells lacking OATP1B1, consistent with molecular docking analyses indicating an overlapping binding orientation on OATP1B1 with the known substrate estrone-3-sulfate. In addition, the liver-to-plasma ratio of pazopanib *in vivo* was decreased in mice with a deficiency of the orthologous transporters, and this was accompanied by diminished pazopanib-induced hepatotoxicity, as determined by changes in the levels of liver transaminases. Our study supports the utility of CCF assays to assess substrate affinity for OATP1B1 within a large set of agents in the class of TKIs and sheds light on the mechanism by which these agents are taken up into hepatocytes in advance of metabolism.

**Significance:**

Despite the established exposure–pharmacodynamic relationships for many TKIs, the mechanisms underlying the agents’ unpredictable pharmacokinetic profiles remain poorly understood. We report here that the disposition of many TKIs depends on hepatic transport by OATP1B1, a process that has toxicologic ramifications for agents that are associated with hepatotoxicity.

## Introduction

The number of FDA-approved small-molecule tyrosine kinase inhibitors (TKI) has rapidly increased since the approval of imatinib in 2001 (1). With more than 60 approved drugs, TKIs have transformed the landscape of modern medicine, especially oncology, with TKIs being critical to the successful treatment of several different cancers (2, 3). Historically, anticancer therapies were developed for intravenous administration, which would be given as inpatient care (4). TKIs represented a paradigm shift, as these drugs can be administered orally and given over prolonged periods of time in an outpatient setting. Although oral dosing offers convenience to patients, it also entails a heightened risk of interindividual pharmacokinetic variability, partially attributable to the limited oral bioavailability of these agents (5) and to drug–drug interactions stemming from polypharmacy (6, 7). Despite the assumption that TKIs would have a milder toxicity profile than cytotoxic chemotherapeutics because of their selective target engagement, these agents may induce severe adverse effects in patients (8, 9), including QTc prolongation, sudden cardiac death, arterial thrombosis, and hepatotoxicity (10); these adverse events may be dose-limiting and frequently necessitate discontinuation of an otherwise effective therapy (9).

Similar to observations made with classic cytotoxic chemotherapeutics (11, 12), the initiation of adverse events associated with TKIs likely relies, at least in part, on drug uptake into injurious sites by xenobiotic transporters. Consequently, a comprehensive understanding of the specific transporters responsible for mediating the membrane crossing of TKIs is essential to predict and understand TKI-induced toxicities. Transport mechanisms for TKIs have previously been evaluated on an individual basis utilizing a variety of methods, often involving the use of radiolabeled drugs (13), fluorescent probes (14), and/or mass spectrometric methods (15). However, the implementation of such nonstandardized techniques has given rise to discrepancies in identifying specific proteins involved in the transport of TKIs. Experimental challenges in traditional, direct uptake assays, in particular the existence of substantial nonspecific, extracellular membrane binding, have limited the ability to reliably and systemically evaluate the mechanisms by which TKIs are taken up into cells (Supplementary Fig. S1A–S1C; ref. 14). Although it is technically possible to overcome artifacts associated with nonspecific binding by separating the outer membrane from the intracellular fraction in direct uptake assays (16), this procedure is cost- and labor-intensive and is not considered feasible for large-scale experiments (17). The utilization of competitive counterflow (CCF) assays presents a viable option for assessing whether a drug is a substrate for a given transporter, as it offers an indirect readout of a probe compound independent of nonspecific binding effects and directly compares substrate affinity for membrane transporters (18). In the present study, we used a CCF assay to screen FDA-approved TKIs in cells that overexpress the organic anion–transporting polypeptide OATP1B1, a transporter that is highly expressed on the basolateral membrane of hepatocytes (19–22) and mediates the hepatic uptake of various, structurally diverse endogenous and xenobiotic compounds (19–22). Our study supports the utility of CCF assays to assess substrate affinity for OATP1B1 with a large set of agents and sheds light on the mechanism by which TKIs are taken up into hepatocytes in advance of metabolism.

## Materials and Methods

### Uptake assays

Cellular accumulation assays were performed in human embryonic kidney cells (HEK293; RRID: CVCL_0045) that were genetically engineered to overexpress human OATP1B1 (23–25). Cell lines were authenticated by Applied Biosystems AmpFISTR Identifiler testing with PCR amplification. Cells were cultured and grown in DMEM supplemented with 10% FBS and maintained at 37°C with 5% CO_2_ (23, 24). All cells were used within passage 30 and verified to be *Mycoplasma*-free using the MycoAlert Mycoplasma Detection Kit (Lonza). At 80% to 90% confluency, cells were seeded on poly-D-lysine–coated 96-well plates 24 hours prior to the assay (2,500 cells/well), and expression of OATP1B1 was induced with 1 μg/mL doxycycline in phenol red–free DMEM. Transport function was assessed using a radiolabeled prototypical substrate for OATP1B1, estradiol [6,7-^3^H(N)]-17β-D-glucuronide (EβG; specific activity, 50 Ci/mmol; purity, 99%; American Radiolabeled Chemicals).

### CCF assays

The CCF assay uses OATP1B1-overexpressing HEK293 cells seeded and cultured as described above. During the study, cells were preincubated at room temperature (∼ 20°C) with 0.01 μmol/L EβG in prewarmed serum-free and phenol red–free DMEM for 1 hour. Following preincubation, each well was spiked with 1 μL of a stock solution containing 0.1, 1, or 10 mmol/L of positive control (EβG), negative control (glucose), test compound dissolved in Dimethylsulfoxide (DMSO), or DMSO only as a vehicle control. Following a 30-minute after incubation with a test compound, the assay was stopped by three consecutive washes with PBS at 4°C. Finally, cells were solubilized with 150 μL of 1% Triton X-100 in PBS for 2 hours at room temperature under constant agitation. A volume of 100 μL of cell lysate was transferred to clear-bottom 96-well isoplates, 200 μL of MicroScint-PS (PerkinElmer) scintillation fluid was added to each sample-containing well, and the plate was subsequently vortex-mixed for 30 seconds. Total radioactivity was determined on a MicroBeta microplate scintillation counter (PerkinElmer), and the resulting counts were then normalized to total protein as determined using a Pierce protein assay (Thermo Fisher Scientific).

### Immunoprecipitation and kinase assay

Immunoprecipitation (IP)-based kinase assays were performed using a modification of a previously reported method (24, 26). Briefly, OATP1B1 overexpressing– or empty vector–containing HEK293 cells were transiently transfected with a Flag-tagged VEGFR plasmid. These cells were then treated with pazopanib (10 μmol/L) or vehicle control. Cell lysates collected in a modified RIPA buffer were then used for Flag-IP as described previously (26). For murine studies, hepatocytes isolated from FVB mice were seeded at a density of 1 × 10^6^ per well and incubated overnight, treated with pazopanib (10 μmol/L) or vehicle control for 1 hour, and then collected for IP. For the *in vitro* kinase assays, myelin basic protein (Active Motif, 31314) was utilized as a prototypical substrate for both serine/threonine and tyrosine kinases to enable *in vitro* kinase assays because of the presence of multiple sites for phosphorylation. These two components were incubated in kinase buffer (Cell Signaling Technology, 9802) supplemented with or without ATP (50 μmol/L) for 30 minutes at 30°C, followed by kinase assays run using the ADP-Glo Kinase Assay kit (Promega). Relative kinase activity was calculated as compared with the vector-transfected cells.

### Molecular docking

The human OATP1B1 structures (PDB code: 8HNB, 8HNC, 8HNH, 8K6L, 8HND, and 8PHW), either in apo form or in complexes with different substrates, were obtained from RCSB PDB (https://www.rcsb.org/). Fabs and cholesterol hemisuccinate in 8PHW were removed. Proteins were prepared and minimized using Protein Preparation Wizard of Schrödinger 2020 (RRID: SCR_016749; Schrödinger, LLC, 2020.) in the OPLS3e force field. Grid was generated by centering on the original ligands or three conservative hydrophobic residues (for apo form 8HNB) with ligand diameter midpoint set to 30 Å. TKIs were prepared using the LigPrep wizard (RRID: SCR_014879) in the same force field followed by Glide SP docking to all the conformation states of OATP1B1 (27). The docking pose with the best docking score was selected.

### Hepatocyte isolation

Mice were euthanized via CO_2_ asphyxiation, and death was confirmed by cardiac puncture. Livers were dissected and stored in PBS supplemented with 1% penicillin–streptomycin and subsequently rinsed three times with PBS in a sterile tissue culture hood. In a Petri dish containing Hank’s Balanced Salt Solution (HBSS) with 0.5 mmol/L EGTA, livers were mechanically dissociated with scissors. This tissue homogenate was centrifuged at 30 × *g* for 5 minutes at 4°C in a 50-mL conical tube. The supernatant was removed, and 10 mL of HBSS was added to the tube. The resuspended liver homogenate was centrifuged again at 30 × *g* for 5 minutes at 4°C, and the supernatant was subsequently removed. To the liver homogenate, 10 mL of 0.05 type IV collagenase was added in HBSS containing 10 mmol/L CaCl_2_. This was incubated at 37°C for 20 minutes under agitation. Next, 25 mL complete medium was added following this incubation to stop enzymatic digestion. Cells were triturated via 25-mL serological pipette to resuspend and immediately filtered through a 100-μm sieve. The cell suspension was centrifuged at 30 × *g* for 5 minutes at 4°C, and the supernatant was discarded. The cell pellet was resuspended with 12 mL complete medium and added to another 50-mL conical tube containing 10 mL 35% Percoll solution in HBSS. Cells were centrifuged at 110 × *g* for 15 minutes, with no braking of the centrifuge. The top most layer of media was discarded, whereas the hepatocyte pellet remained undisturbed. Next, 30 mL of complete media was added, and hepatocytes were resuspended. Purified hepatocytes were centrifuged again at 30 × *g* for 5 minutes, and the supernatant was discarded. Hepatocytes were resuspended in 10 mL of complete media, seeded 24 to 48 hours prior to experimentation, and incubated at 37°C with 5% CO_2_.

### Murine pharmacokinetic studies

For pharmacokinetic studies, plasma and liver tissue samples were collected from male wild-type (WT) mice (8–13 weeks old) and sex- and age‐matched OATP1A/1B cluster–knockout (OATP1A/1B-KO) mice following an established protocol (28). This model provides a translationally useful model of an OATP1B1-deficient phenotype that is insensitive to compensatory mechanisms associated with redundant OATP1A-type transporters in mice. All animals were on an FVB background strain, were given a standard diet and water ad libitum, and were housed and handled in accordance with the Institutional Animal Care and Use Committee at The Ohio State University (protocol #2015A00000101-R2). Pazopanib was administered to mice as a single oral dose (300 mg/kg) dissolved in sterile PBS as described (29) after a 3-hour fasting. Serial blood samples were collected in accordance with a previously established protocol (28). At the terminal timepoint, the mice were euthanized via carbon dioxide inhalation, and the blood was collected by cardiac puncture with a needle and 1-mL syringe and transferred to a 1.5-mL heparinized tube. Next, the samples were centrifuged at 15,000 × *g* for 5 minutes, and the plasma supernatant was collected. All samples were analyzed at the Laboratory of Translational Pharmacology (Erasmus MC Cancer Institute, Rotterdam, the Netherlands) for the presence of pazopanib by a validated analytical method based on LC/MS-MS (30). Pharmacokinetic parameters were calculated by noncompartmental methods using Phoenix WinNonlin (RRID: SCR_024504) version 8.2 (Certara).

### Data availability

The data generated in this study are available within the article and its Supplementary Data files. Experimental results from uptake studies were normalized to total protein content and baseline values and expressed as a percentage. An unpaired two-sided Student *t* test with Welch correction or a two-way ANOVA was used to compare group differences. Values are presented as mean ± SD unless stated otherwise in the figure captions. A cutoff value of *P* < 0.05 was used for statistical significance, and all analyses were performed using the software package Prism 9 (GraphPad).

## Results

### Optimization and validation of CCF assays for OATP1B1 substrates

**Figure 1:**
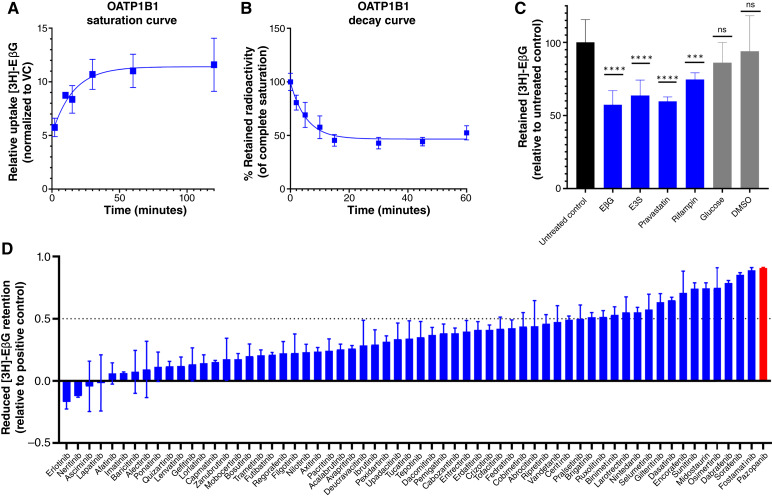
Development and validation of the CCF method. **A,** Uptake of 0.01 μmol/L [^3^H]-EβG in OATP1B1-overexpressing HEK293 cells was evaluated at room temperature over 120 minutes in 96-well plates (*n* = 4 technical replicates, representative of *n* = 2 biological replicates; error bars represent SD). **B,** After saturation of cells at 1 hour, 100 μmol/L unlabeled EβG was spiked into wells, causing efflux of [^3^H]-EβG (*n* = 6 technical replicates across *n* = 2 biological replicates; error bars represent SD). **C,** After 1 hour of saturation with 0.01 μmol/L [^3^H]-EβG, 100 μmol/L positive (blue bars) and negative (gray bars) control substrates of OATP1B1 were spiked into wells and incubated for 30 minutes. Final intracellular radioactivity was measured and presented as relative to untreated control (*n* = 9 technical replicates across *n* = 3 biological replicates; error bars represent SD. ***, *P* < 0.001; ****, *P* < 0.0001; five compared with the control). **D,** Stimulated efflux of preloaded [^3^H]-EβG stimulated after the addition of 10 μmol/L TKI. Final intracellular radioactivity was measured and presented as relative to the efflux induced by an equimolar concentration of EβG, a known substrate and efflux inducer (*n* = 6 technical replicates across *n* = 2 biological replicates; error bars represent SEM). VC, vector control.

In order to establish an effective CCF assay, steady-state equilibrium conditions need to be established for the transporter (18). To ascertain the saturation time for OATP1B1-overexpressing cells, we conducted a 3-hour time course assay in standard 96-well plates using EβG (0.01 μmol/L) as a prototypical OATP1B1 substrate (Fig. 1A). The intracellular levels reached equilibrium in OATP1B1-overexpressing cells at 45 minutes, as evidenced by an initial plateau in radioactivity readings over time, which was maintained for at least 120 minutes. Consequently, a 1-hour preincubation was used for all subsequent experiments to achieve steady-state conditions. Similarly, the time-dependent efflux of EβG was characterized by spiking unlabeled EβG at a concentration of 100 μmol/L 1 hour after the initial preincubation with radiolabeled EβG, resulting in a second steady state being observed at 30 minutes (Fig. 1B). Therefore, 30 minutes was chosen as an adequate coincubation time to induce counterflow and stimulated efflux for all following assays.

To further validate this CCF assay for OATP1B1 and ensure its independence from EβG-specific effects, multiple established transported substrates of OATP1B1 were tested as efflux inducers at excess concentrations of 100 μmol/L; EβG (positive control), estrone-3-sulfate (E3S), pravastatin, and rifampin all induced significant efflux of the preloaded radiolabeled EβG under the described conditions (Fig. 1C). Conversely, glucose, which is not a substrate of OATP1B1 and DMSO, used as a vehicle control, failed to induce efflux, indicating that the developed method constitutes a valid, OATP1B1 substrate–specific initiation of efflux.

### Evaluation of TKIs as OATP1B1 substrates using CCF assays

We next evaluated FDA-approved TKIs as potential substrates of OATP1B1 utilizing the CCF assay, comprising a set of 62 compounds (Fig. 1D). To ensure proper functioning of the cells and assay, EβG was included as a positive control in all studies. Despite previous reports that suggest 10 times the IC_50_ of a test compound as the optimal concentration for counterflow evaluation (31), we opted to evaluate final experimental concentrations of 1, 10, and 100 μmol/L of the test compounds because of the scale of the screen, potential solubility constraints of some of the test compounds, and the ability to reliably identify putative substrates of OATP1B1 (Fig. 1D; Supplementary Fig. 2SA and S2B; refs. 18, 31).

In order to correlate radiolabeled EβG efflux with the ability of a given test compound to be itself transported by OATP1B1, we normalized the final intracellular radioactivity of cells treated with each TKI to those cells treated with an equimolar concentration of the positive control (unlabeled EβG). Therefore, a reported CCF value of 1 indicates an efflux equal to that initiated by EβG, <1 indicates efflux less than that initiated by EβG, and >1 indicates efflux greater than that initiated by EβG. We set an arbitrary threshold of 0.5 (or 50% efflux relative to EβG) to identify potential substrates of OATP1B1 in the set of TKIs. Our screening results identified several TKIs as putative substrates of OATP1B1 (Supplementary Table S2). It is important to note that various patterns of efflux were noted when examining potential concentration dependence. For some TKIs (ceritinib, cobimetinib, crizotinib, erdafitinib, nintedanib, osimertinib, sunitinib, tofacitinib, and vandetanib), efflux was clearly dependent on concentration, and a few TKIs only induced efflux at the highest concentration tested. Additionally, a subset of TKIs (axitinib, cabozantinib, dabrafenib, dasatinib, futibatinib, lapatinib, nilotinib, pexidartinib, pralsetinib, selumetinib, and tucatinib) exhibited an unexpected efflux pattern solely at lower concentrations, which can potentially be attributed to cytotoxic effects of high concentrations of these agents on cells during the incubation period. Among the TKIs identified as substrates, as indicated by the induction of efflux by more than 50% compared with the cold EβG as control, across the tested concentration range, 16 compounds induced efflux by >50% at 1 μmol/L, 16 compounds at 10 μmol/L, and 13 compounds at 100 μmol/L (Supplementary Table S2).

### Validation studies of pazopanib as an OATP1B1 substrate

**Figure 2:**
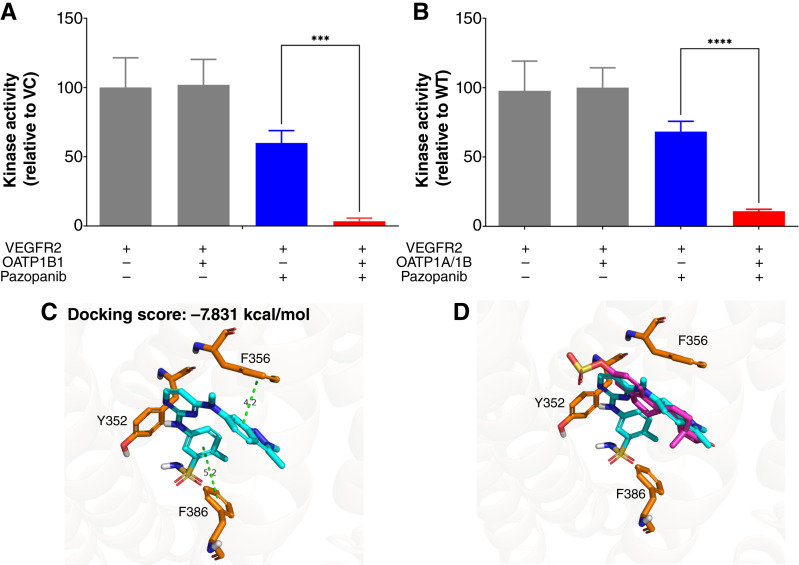
Using a biomarker and *in silico* docking to evaluate pazopanib as a substrate. Kinase activity of VEGFR kinase from OATP1B1-overexpressing and vector control (VC) cells (**A**) and hepatocytes isolated from WT and OATP1A/1B-KO mice (error bars represent SD; ***, *P* < 0.001; **B**) following a 1-hour treatment with 10 μmol/L pazopanib. Kinase activity was assessed by ADP-Glo (error bars represent SD; ****, *P* < 0.0001). Molecular *in silico* docking of pazopanib in elucidated OATP1B1 structure (**C**) and with E3S superimposed (**D**).

Based on the results of the CCF assays at all concentrations and its association with toxicodynamic events originating in the liver (32–35), pazopanib was chosen as a representative compound for further validation. To substantiate the findings from the CCF assay, we employed a kinase assay to assess the extent of pazopanib influx mediated by OATP1B1 into cells. Cells that overexpressed OATP1B1 or the corresponding vectors were transfected to express VEGFR2 , the primary target kinase for pazopanib (36). These cells were then treated with pazopanib, the kinase was collected using immunoprecipitation, and a kinase assay was carried out. OATP1B1-overexpressing cells exposed to pazopanib exhibited a markedly reduced kinase activity compared with control cells that did not overexpress OATP1B1 (Fig. 2A). This suggests that pazopanib was able to exert a greater pharmacodynamic effect in the presence of OATP1B1, implying a greater accumulation of pazopanib in these cells. Similarly, pazopanib caused a greater decrease in kinase activity in hepatocytes from WT mice relative to those from mice lacking the orthologous transporters (OATP1A/1B-KO mice). This implies that pazopanib is transported into hepatocytes by OATP1A- and/or OATP1B-type transporters natively expressed in murine hepatocytes (Fig. 2B).

In addition to kinase activity, the results of an *in silico* molecular docking study provided additional validation and characterization of pazopanib’s interaction with OATP1B1. In this analysis, pazopanib docks best to 8PHW, which occupies the substrate binding pocket of OATP1B1 in an orientation resembling E3S, a known, experimentally verified substrate (Fig. 2C and D). The docking score of pazopanib to 8PHW (−7.8 kcal/mol; Supplementary Tables S3 and S4) is comparable to that of E3S to 8PHW (−8.0 kcal/mol). No specific polar contacts were observed; instead, binding relied on hydrophobic contacts and π-π stacking interactions with adjacent residues, including Phe356, Tyr352, and Phe386. Taken together, these results provide further confirmation that pazopanib is a transported substrate of OATP1B1. To further understand physicochemical characteristics that may drive the ability of OATP1B1 to transporter TKIs, similar docking studies were performed for all TKIs included in the CCF screens (Supplementary Tables S3 and S4). Interestingly, all TKIs identified as substrates in the CCF assay, at any tested concentration, could be successfully docked into OATP1B1, and all but one interacted most favorably with the minor pocket–open state of the transporter. Furthermore, of the TKIs not identified as a substrate of OATP1B1, a majority of them failed to be docked successfully, and the TKIs that did show an interaction with OATP1B1 in the CCF assay had significantly worse docking scores than the identified positive hits.

**Figure 3:**
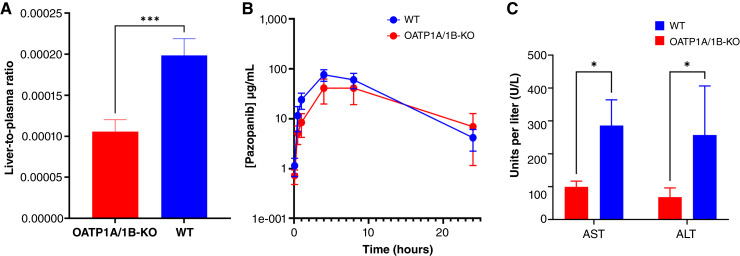
Pharmacokinetics, tissue accumulation, and serum chemistry following pazopanib administration. **A,** Liver accumulation of pazopanib in WT (blue) or OATP1A/1B-KO mice (red) normalized to the plasma concentration of pazopanib at 24 hours (*n* = 5; error bars represent SD; ***, *P* < 0.001). **B,** Mean plasma concentration time profile of pazopanib in WT (blue) or OATP1A/1B-KO mice (red) after oral administration of pazopanib at a dose of 300 mg/kg (*n* = 5 technical replicates; error bars represent SD). **C,** Serum levels of AST and ALT 24 hours after a single 300 mg/kg oral dose of pazopanib in WT (blue) or OATP1A/1B-KO mice (red; *n* = 4; error bars represent SD; *, *P* < 0.05).

The documented occurrence of severe and potentially fatal hepatotoxicity associated with pazopanib administration prompted the inclusion of a black box warning in its prescribing information (32). We speculated that the occurrence of this side effect may be dependent on OATP1B-type transporters, based on the general thesis that cell-specific expression of transporters serves as a mechanism governing the uptake of toxic drugs for a selective injury to targeted cells. Based on prior findings (29) demonstrating the onset of acute hepatotoxicity in mice 24 hours postadministration of a single 300 mg/kg oral dose of pazopanib, we administered an equivalent dose to both WT mice and OATP1A/1B-KO mice. The accumulation of pazopanib in the livers of WT mice was significantly higher after 24 hours compared with the levels observed in OATP1A/1B-KO mice, suggesting that OATP1A/1B transporters are responsible, at least in part, for the uptake of pazopanib into hepatocytes (Fig. 3A). The observed changes in the accumulation and hepatic distribution of pazopanib were not accompanied by proportional changes in drug levels in plasma, further substantiating the notion that changes in liver uptake were due to a transporter defect, and not to alterations in systemic exposure (Fig. 3B; Supplementary Table S5). As biomarkers of liver injury, aspartate transaminase (AST) and alanine transaminase (ALT) were quantified in plasma 24 hours after pazopanib administration. Following exposure to pazopanib, WT mice experienced significantly increased levels in circulating AST and ALT compared with levels observed in OATP1A/1B-KO mice, implying that the presence of OATP1A/1B is necessary to induce the recorded toxicity phenotypes (Fig. 3C).

## Discussion

In recent years, CCF assays have proven to be a reliable method for identifying potential substrates of a xenobiotic transporter using fluorescent or radiolabeled probes (18, 37–41). Our investigation adds to this field by systematically assessing FDA-approved TKIs as putative substrates of OATP1B1. This method circumvents methodologic issues associated with nonspecific extracellular membrane binding that would otherwise confound the results of a traditional, direct uptake, and it thus allows for a more reliable result for those compounds that cause artificial results in a direct uptake assay. The positive and negative controls acted as expected during the assay validation, and we identified 13 TKIs (of 62) as potential substrates of OATP1B1 across a range of concentrations. Among the hits, several TKIs have previously been claimed to be substrates of OATP1B1 (42), including axitinib, crizotinib, pazopanib, and sorafenib, and several other TKIs have been reported to inhibit OATP1B1, including encorafenib, midostaurin, pazopanib, and pralsetinib. Interestingly, about half of the identified substrates are marketed TKIs that include warnings and precautions in the prescribing information for hepatotoxicity. This supports the possibility that hepatic uptake mechanisms for these agents depend on OATP1B-type transporters (Supplementary Table S1).

Fostamatinib, midostaurin, pazopanib, and sorafenib were hits in our CCF assay at all three tested concentrations. Of these four compounds, fostamatinib is a known hepatotoxin for which OATP1B1-dependent interactions have not been documented before, either in the literature or in its product label. Similarly, midostaurin has not previously been recognized as a substrate for this transporter, although the agent is a known potent inhibitor of both OATP1B1 (IC_50_ = 0.3–1.3 μmol/L) and the related liver transporter OATP1B3 (IC_50_ = 5 μmol/L; ref. 43). In contrast, both pazopanib and sorafenib have been reported as substrates of the OATP1B1, in both regulatory documents and by independent, academic investigations (13, 44).

It is important to note that there are observed deviations in expected concentration-dependent efflux patterns within the 13 lead compounds identified in our study, and such patterns have been reported in other counterflow-type methods (18). Reasons for this and other limitations of this method include the fact that (i) high concentrations of test compounds can reduce cellular viability over the course of loading and efflux, causing a lack of expected cellular efflux at the higher range when it was already observed at the lower concentrations; (ii) a TKI may have relatively weak affinity for OATP1B1 such that interactions in a CCF assay may not be observed at lower concentrations and induction of efflux occurs only at higher concentrations; and (iii) solubility limitations at high test concentrations may compromise interactions with the transporter and the TKI. In addition, to address those compounds that slightly, but not significantly, induced probe uptake (as indicated by a CCF value of <0), it is possible that inhibition of an efflux transporter expressed natively in HEK293 cells may effectively sequester the EβG probe within the cell, as they are integral in the establishment of a steady-state equilibrium causing a deceptive “increased uptake” of the probe following TKI spike. Given these limitations, although our positive hits reliably exhibit previously identified substrate characteristics of OATP1B1, the absence of certain agents among these hits does not preclude them from being considered as potential substrates (14, 45–52). However, traditional uptake assays have limitations that necessitate the use of indirect methods, such as the time-consuming and costly nature of establishing reliable transport kinetics or separating the membrane and intracellular fractions to eliminate the effects of nonspecific binding; nonspecific membrane binding is a known issue with cellular uptake assays and limits our ability to perform uptake assays with select compounds. A hallmark of nonspecific membrane binding is the drastically increased signal in both control cells and transporter-expressing cells; this decreases the signal-to-noise ratio to a point at which changes associated with the expression of a transporter become indiscernible (Supplementary Fig. S1A–S1C). The CCF method employed in the present studies avoids these obstacles and is easily scalable to 96-well plates with minimal wash steps, thereby introducing a cost- and time-effective advantage in facilitating these types of studies.

In confirmatory studies, pazopanib emerged as a compelling candidate because of its consistent identification as a substrate across all three tested concentrations. *In silico*, pazopanib exhibited strong characteristics of an OATP1B1 substrate. Indeed, molecular docking of pazopanib into the recently elucidated structure of OATP1B1 (27) showed thermodynamically favorable interactions with the binding pocket, mediated predominantly by hydrophobic interactions and π-π stacking with nearby phenylalanine residues. This is further substantiated by the superposition of pazopanib and other known OATP1B1 substrates, demonstrating the steric similarities of binding and achieving comparable docking scores. Specifically, pazopanib preferably interacts with the minor pocket–open state of OATP1B1, which is constructed by the three hydrophobic residues Y352, F356, and F386 (53). Furthermore, docking all positive hits from any concentration showed successful and preferred interaction with this configuration, suggesting a potential driving factor that renders TKIs substrates of OATP1B1. Shape complementarity to the minor pocket and interaction with the hydrophobic residues that form this pocket may be an important factor to select substrates for the OATP1B1 transporter. The computational model of pazopanib successfully interacting with the binding pocket of OATP1B1 provides further support for the thesis that this TKI acts as a transported substrate.

As further confirmation of intracellular translocation of pazopanib by OATP1B1, we exploited its kinase inhibition activity as a surrogate signal for intracellular transport with a biosensor. After transfection with VEGFR, cells proficient (OATP1B1-overexpressing HEK293 cells and hepatocytes isolated from WT mice) or deficient for OATP1B-type transporter proteins (vector control HEK293 cells and hepatocytes isolated from OATP1A/1B-KO mice) were coincubated with pazopanib. These studies support the thesis that both *in vitro* and *ex vivo*, OATP1B-type transporters are integral to the cellular uptake of pazopanib. Interestingly, the partial decrease in kinase activity following exposure to pazopanib in OATP1B-deficient cells suggests the involvement of one or more additional uptake transporters in the cellular uptake of pazopanib, and these may include organic cation transporter OCT1, which is also abundantly expressed on mammalian hepatocytes (54).

A potentially interesting connection of the transport of TKIs with OATP1B1 is in the context of pazopanib-induced hepatotoxicity, a serious health concern in both clinical and preclinical settings (33, 34). The mechanisms underlying pazopanib-induced hepatotoxicity remain to be completely elucidated. Our findings point to the possibility that the transport of pazopanib into hepatocytes is a necessary initiating event that precedes the observed toxicity. Although the translational relevance of this thesis requires further exploration in future studies, this screen serves as proof of principal that OATP1B-type transporters may be a therapeutic target to prevent or ameliorate pazopanib-induced liver injury. This possibility is consistent with the finding that following a hepatotoxic dose of pazopanib, relatively greater increases in the liver enzymes AST and ALT were observed in WT mice compared with OATP1A/1B-KO mice and that these observations are in line with the decreased concentrations of pazopanib in the liver of animals engineered to be deficient for OATP1A/1B transport. It is tempting to speculate that this mechanism may have clinical significance for the future development of combination therapies involving TKIs that are transported by OATP1B1 and for the evaluation of pharmacokinetic drug–drug interactions. Indeed, modulation of OATP1A/1B-mediated transport may alter the liver accumulation of pazopanib, or other transported TKI substrates, and may either increase or decrease the susceptibility to the development of drug-induced toxicities. Interestingly, the diminished liver accumulation in OATP1A/1B-KO mice occurred without statistically significant changes in the exposure of pazopanib in plasma. Although this observation is not unprecedented and has previously been documented for other OATP1B1 substrates, such as the taxanes paclitaxel and docetaxel as well as the vinca alkaloid vincristine (12, 55), it is possible that hepatic uptake is not a rate-limiting step in pazopanib elimination and/or that this distribution process is not exclusively contingent on a single uptake transporter.

### Conclusion

In this study, we established and validated a CCF assay using radiolabeled EβG in OATP1B1-overexpressing HEK293 cells; this assay was then used to evaluate the transport mechanisms of a set of FDA-approved TKIs. Our findings demonstrate the importance of OATP1B1 as an important mediator of the cellular uptake of select TKIs, suggesting that this transport mechanism contributes to the hepatic uptake and elimination of these agents. In view of the established exposure–toxicity relationships for many TKIs, the observations made here further suggest that OATP1B1-mediated transport, which occurs in advance of hepatic metabolism, may contribute to interindividual pharmacokinetic variability observed previously in patients with cancer requiring treatment with TKIs. Collectively, these findings confirm the utility of CCF screening approaches for identifying substrates of this clinically important xenobiotic transporter and may assist in understanding the mechanisms underlying drug–drug interactions and toxicities for TKIs identified as OATP1B1 substrates.

## Supplementary Material

Supplementary Data SI Text and Figure Legends

Supplementary Figure 1 Figure S1. In Vitro Uptake

Supplementary Figure 2 Figure S2. CCF at 1 & 100 uM

Supplementary Table 1 Table S1. List of Tyrosine Kinase Inhibitors and OATP1B-type Transporter interactions

Supplementary Table 2 Table S2. Table of Hits

Supplementary Table 3 Table S3. Molecular Docking Positive

Supplementary Table 4 Table S4. Molecular Docking Negative

Supplementary Table 5 Table S5. PK Parameters
