# Supplementary material for: Systematic Evaluation of Tyrosine Kinase Inhibitors as OATP1B1 Substrates Using a Competitive Counterflow Screen
Source: Cancer Res Commun. 2024 Sep 23;4(9):2489–97. doi: 10.1158/2767-9764.CRC-24-0332 (PMC11417675; doi:10.1158/2767-9764.CRC-24-0332)
Supplement: Supplementary Data — SI Text and Figure Legends [file crc-24-0332_supplementary_data_suppsd1.docx]

Research Article - Cancer Research Communications

**Systematic Evaluation of Tyrosine Kinase Inhibitors as OATP1B1 Substrates Using a Competitive Counterflow Screen**

Thomas Drabison^1^, Mike Boeckman^1^, Yan Yang^2^, Kevin M. Huang^1^, Peter de Bruijn^3^, Mahesh R. Nepal^1^, Josie A. Silvaroli^1^, Anika T. Chowdhury^1^, Eric D. Eisenmann^1^, Xiaolin Cheng^2^, Navjotsingh Pabla^1^, Ron H. J. Mathijssen^3^, Sharyn D. Baker^1^, Shuiying Hu^1^, Alex Sparreboom^1^, and Zahra Talebi^1^

**Authors’ Affiliations:** ^1^Division of Pharmaceutics and Pharmacology, College of Pharmacy & Comprehensive Cancer Center, The Ohio State University, Columbus, Ohio; ^2^Division of Medicinal Chemistry and Pharmacognosy, College of Pharmacy, The Ohio State University, Columbus, Ohio; ^3^Department of Medical Oncology, Erasmus MC Cancer Institute, University Medical Center Rotterdam, The Netherlands

**Supplemental Methods:**

**Supplemental Figure 1.**

**Traditional methods to evaluate TKI uptake into cells *ex vivo* and *in vitro*.**

**(A)** [^3^H]-EβG (positive control for OATP1A/1B type transporters) uptake evaluated *ex vivo* in hepatocytes isolated from wild-type (blue; WT) or OATP1A/1B-KO mice (red). (n=3, error bars represent SD). **(B)** [^3^H]-Pazopanib uptake evaluated *ex vivo* in hepatocytes isolated from wild-type (blue) or OATP1A/1B-KO mice (red) (n=3, error bars represent SD). Studies were performed in the presence or absence of the OATP1B-type transporter inhibitor, rifampin, at a dose known to affect OATP1B1 function (20 µM). **(C)** [^3^H]-EβG and [^3^H]-pazopanib uptake evaluated *in vitro* in vector control cells (blue; VC) or OATP1B1-overexpressing HEK293 cells (red) (n=3, error bars represent SD).

**Supplemental Figure 2.**

**Evaluation of TKIs as substrates of OATP1B1 at 1μM and 100 μM.**

Stimulated efflux of preloaded [^3^H]-EβG stimulated by the addition of TKIs at concentrations of 1 μM **(A)** and 100 μM **(B)**. Final intracellular radioactivity was measured and presented as relative to the efflux induced by equimolar concentration of EβG, a known substrate and efflux-inducer (n=6 technical replicates across n=2 biological replicates; error bars represent SEM).

**S.I. Table 2:** Tyrosine kinase inhibitors identified as substrate via CCF at 1, 10, and 100 μM.

**S.I. Table 3.**

**Docking Score of TKIs Identified as Substrates to Different PDB Structures of OATP1B1**

**S.I. Table 4.**

**Docking Score of TKIs Not Identified as Substrates to Different PDB Structures of OATP1B1**

**S.I. Table 5.**

**Pharmacokinetic parameters of pazopanib in FVB wild-type or OATP1A/1B Knock-out mice.**

Data represent mean ± SD in parenthesis.

*Abbreviations:* C_max_, observed peak concentration; N, total number of observations; AUC_0-24;_ area under the plasma concentration-time curve.
