# Supplementary figures and images for: Systematic Evaluation of Tyrosine Kinase Inhibitors as OATP1B1 Substrates Using a Competitive Counterflow Screen
Source: Cancer Res Commun. 2024 Sep 23;4(9):2489–97. doi: 10.1158/2767-9764.CRC-24-0332 (PMC11417675; doi:10.1158/2767-9764.CRC-24-0332)

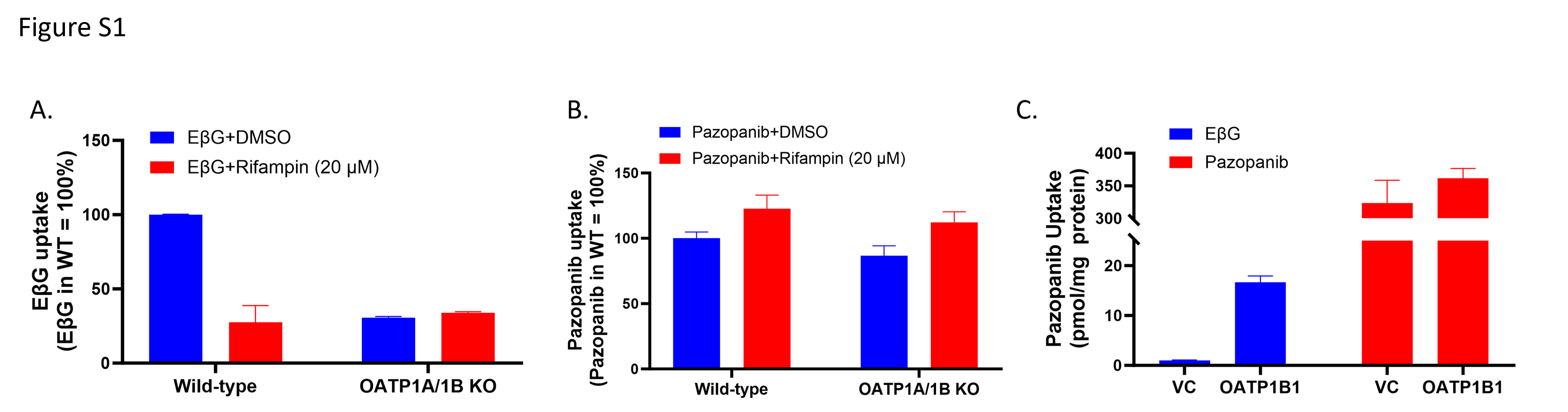

Supplement: Supplementary Figure 1 — Figure S1. In Vitro Uptake [file crc-24-0332_supplementary_figure_1_suppsf1.png]

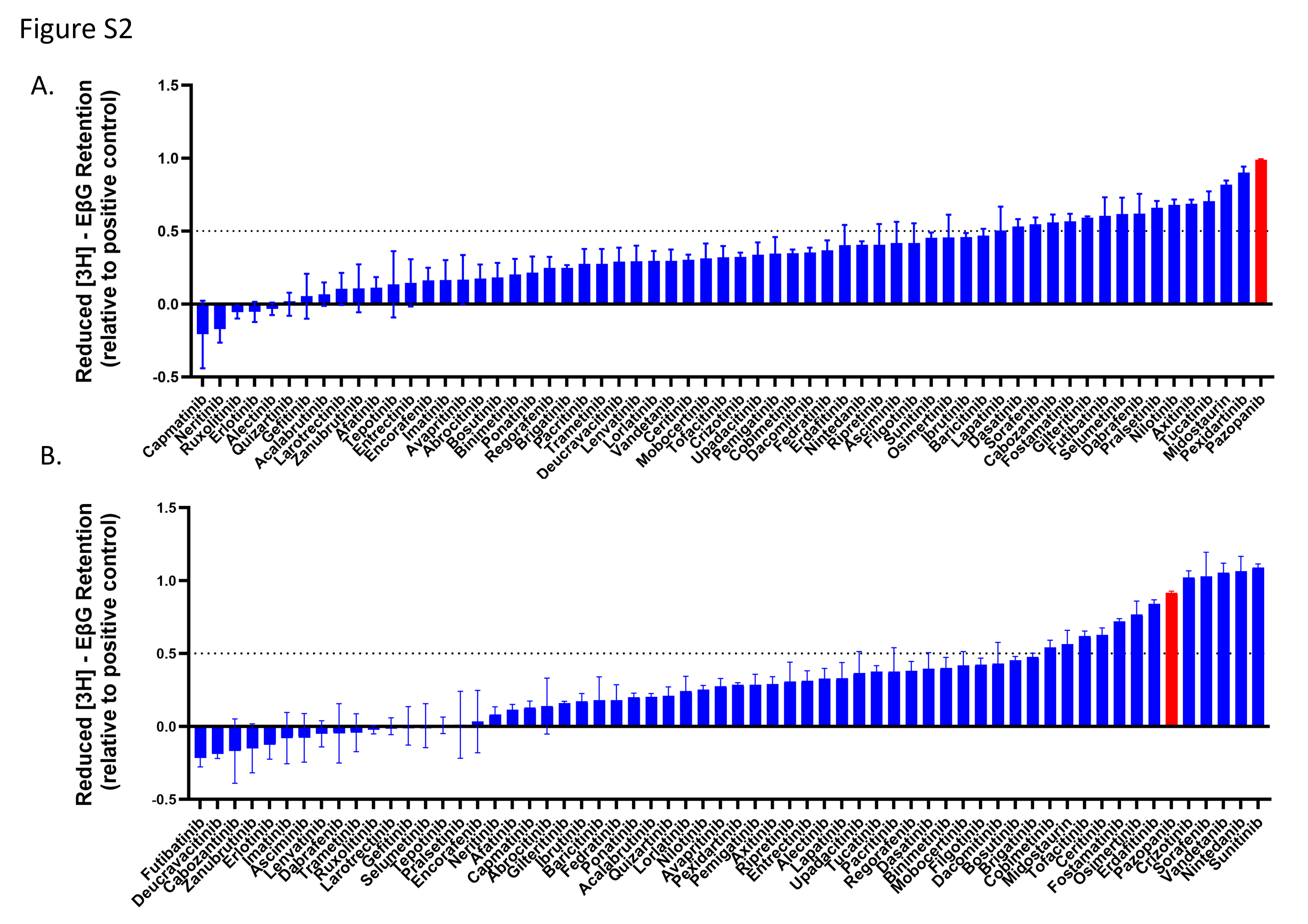

Supplement: Supplementary Figure 2 — Figure S2. CCF at 1 & 100 uM [file crc-24-0332_supplementary_figure_2_suppsf2.png]

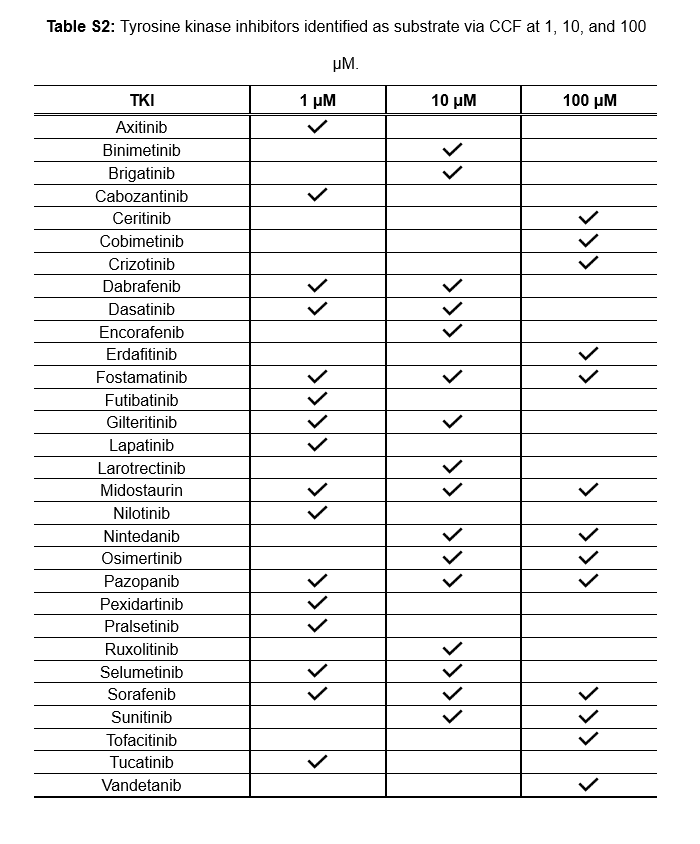

Supplement: Supplementary Table 2 — Table S2. Table of Hits [file crc-24-0332_supplementary_table_2_suppst2.png]

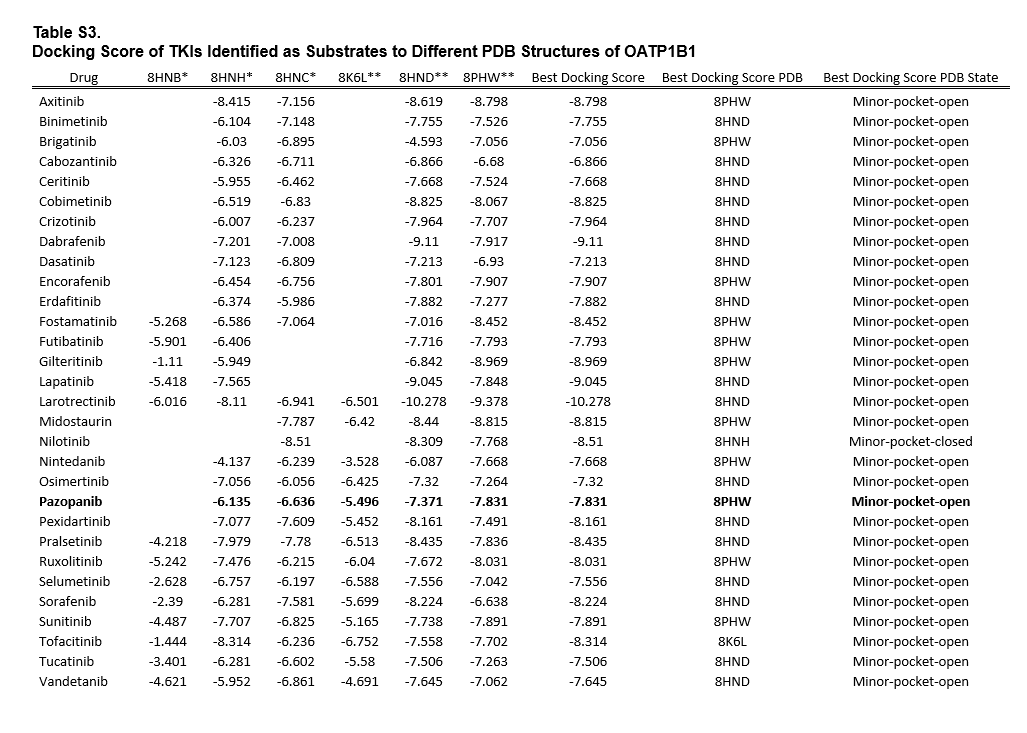

Supplement: Supplementary Table 3 — Table S3. Molecular Docking Positive [file crc-24-0332_supplementary_table_3_suppst3.png]

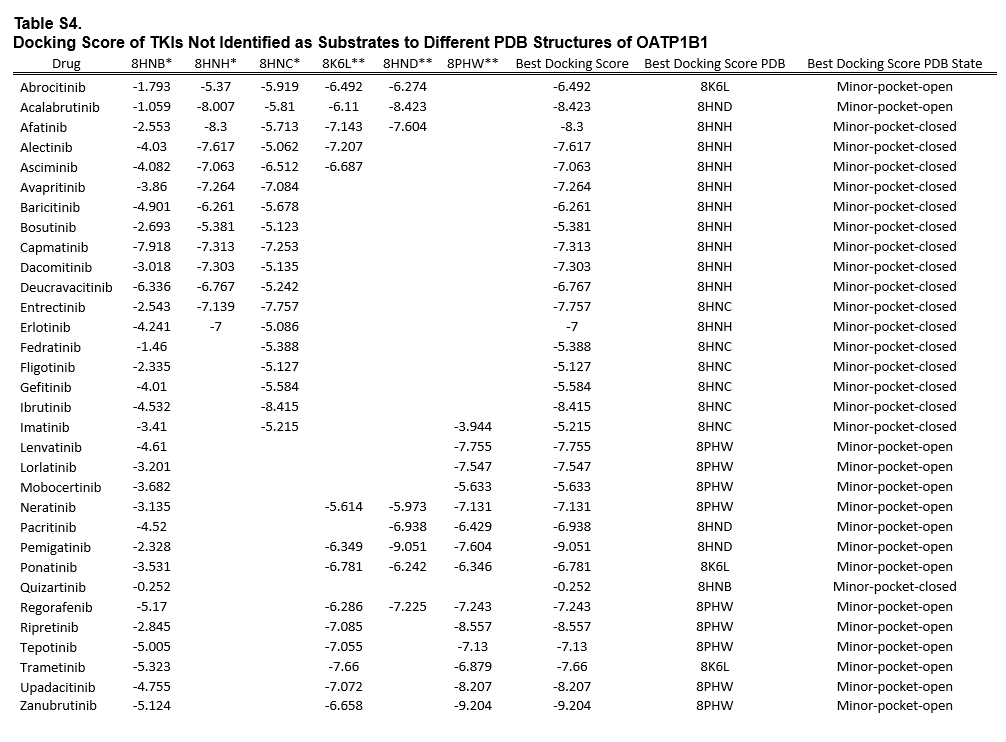

Supplement: Supplementary Table 4 — Table S4. Molecular Docking Negative [file crc-24-0332_supplementary_table_4_suppst4.png]

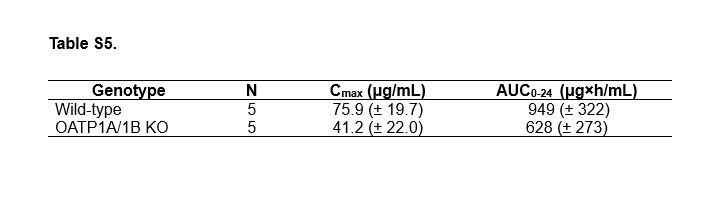

Supplement: Supplementary Table 5 — Table S5. PK Parameters [file crc-24-0332_supplementary_table_5_suppst5.png]
